# Economic evaluation of intravenous iron formulations for patients with iron deficiency anemia: a systematic review

**DOI:** 10.3389/frhs.2025.1690519

**Published:** 2025-11-19

**Authors:** Zhicong Xing, Shengjun Mu, Qingxia Xue, Fudong Sun, Guige Hou, Quan Zhao

**Affiliations:** 1 School of Pharmacy, Binzhou Medical University, Yantai, Shandong, China; 2 Department of Pharmacy, Yantai Yuhuangding Hospital, Yantai, Shandong, China

**Keywords:** intravenous iron formulations, iron deficiency anemia, economic evaluation, health economics, systematic review

## Abstract

**Background:**

Various intravenous iron formulations show great promise in the treatment of iron deficiency anemia (IDA), and economic evaluation results are becoming increasingly important as criteria for allocating healthcare resources. This study aimed to systematically evaluate the economics of six main intravenous iron formulations in the treatment of IDA.

**Methods:**

Computerized search of relevant studies in PubMed, Embase, Web of Science, and The Cochrane Library to collect economic evaluation of six intravenous iron formulations for the treatment of patients with IDA; the time limit for searching was from the establishment of the database to 30 July 2025. Two reviewers independently screened literature, extracted data, evaluated the quality of included studies using the Consolidated Health Economic Evaluations Reporting Standards 2022, and performed descriptive analyses.

**Results:**

Of the 2,288 articles retrieved, 17 studies were included, including five drugs, conducted in 10 different countries. Six studies compared ferric carboxymaltose (FCM) with iron sucrose (IS); two studies compared FCM, ferric derisomaltose (FDI), and IS; one study compared FCM, iron dextran (ID), and IS; one study compared FCM, IS, ID, and FDI at three dose levels; one study obtained an economic ranking for FCM, ID, IS, and ferrous gluconate (FG); five studies compared FDI and FCM; and one study compared FDI with IS. The overall quality of the included studies was high. A total of 13 studies conducted sensitivity analyses to check the robustness of their results.

**Conclusion:**

This review systematically evaluates the economic characteristics of the six main intravenous iron formulations for treating IDA. Current evidence suggests that the efficacy of FDI is better than IS, and the economic ranking of the four intravenous iron formulations can be summarized as FCM, ID, IS, and FG. Further research is needed to justify the economic comparison between FCM and FDI.

## 1 Introduction

Anemia is defined as an insufficient number of red blood cells or a below-average level of hemoglobin (Hb) within these cells (1). The latest epidemiological studies show that approximately 1.92 billion people worldwide suffer from anemia, accounting for 24.3% of the total population (2). The cost of treating anemia involves medications, blood transfusions, hospitalization, and other medical care. In the United States, the cost of medical care for patients with anemia is usually higher than for patients without anemia. For example, among patients with cardiovascular disease, the average annual medical cost for those with anemia was $22,926, compared with $17,189 for those without anemia (3). Over the long term, chronic anemia increases the financial burden on individuals and families and puts pressure on the public health system. Anemia increases medical costs by approximately 1.8 times when co-existing with other diseases such as chronic kidney disease (3). In addition to increased direct medical costs, anemia causes indirect economic losses through reduced labor productivity, increased sick leave, and early deaths (4). This means that anemia imposes a huge financial burden on individuals and the healthcare system.

Iron deficiency anemia (IDA) is the most common type of anemia worldwide (5), accounting for approximately 50% of anemia cases (6, 7). IDA affects multiple organ systems and disease areas, worsening chronic conditions such as heart failure, digestive disorders, chronic kidney disease, and neoplasms in adults (8), leading to adverse perinatal outcomes in pregnant women, and causing growth retardation and cognitive impairment in infants and children (9). It has been shown that the prevalence of iron deficiency or IDA in patients with chronic heart failure, cancer, chronic kidney disease, inflammatory bowel disease (IBD), and pregnant women is approximately 37%–61%, 33%–43%, 24%–85%, 13%–90%, and 66.7%, respectively (8, 10, 11). Thus, the prevention and treatment of IDA have become a noteworthy public health challenge that affects the well-being of individuals and significantly impacts the economic progress of societies and countries (12).

Clinically, for patients with IDA, especially those with comorbid underlying diseases, appropriate iron supplementation should be selected according to the degree and underlying cause of iron deficiency, with oral and intravenous iron formulations being the most commonly used options (13). Oral iron supplements are convenient to use and inexpensive and can be effective in correcting IDA in many patients; however, their efficacy may be limited in cases where ongoing losses exceed intestinal absorption capacity or when gastrointestinal intolerance occurs (14, 15). Intravenous iron formulations provide a route of administration independent of intestinal iron absorption capacity, allowing for rapid correction of anemia and obviating the need for oral iron, thereby eliminating gastrointestinal side effects associated with oral therapy (16). A meta-analysis (17) showed that oral iron supplements have significant gastrointestinal effects (nausea, vomiting, and altered bowel movements), whereas intravenous iron formulations have less common side effects. Intravenous iron formulations have become the recommended first-line treatment for patients who are intolerant or unresponsive to oral iron supplements in national guidelines and consensus (18–22). Therefore, for IDA patients with malabsorption, intolerance, and poor adherence to oral iron supplements (e.g., the elderly, pregnant women, and patients with gastrointestinal disorders) and postoperative patients who require rapid iron supplementation, intravenous iron formulations offer a better alternative (23).

Iron sucrose (IS), ferrous gluconate (FG), iron dextrose (ID), ferumoxytol (FMT), ferric carboxymaltose (FCM), and ferric derisomaltose (FDI) are the six currently marketed intravenous iron formulations. Among them, IS and FG are small-dose intravenous iron formulations. IS was widely used for treating IDA, showing good efficacy and a low incidence of allergic reactions. However, IS requires multiple administrations, which may reduce compliance (24). FG has poor molecular stability, leading to dosage limitations during infusion, requiring longer infusion times and multiple administrations. Additionally, the higher risk of allergic reactions further restricts its widespread use (25). ID has the option of multiple doses or a single total dose infusion. Total dose infusion reduces the number of doses and costs for patients but requires longer infusion time (26). FMT, FCM, and FDI are high-dose intravenous iron formulations. Among currently available formulations, FMT enables rapid administration, but attention should be paid to possible hypersensitivity reactions associated with faster injection rates (27). The maximum single intravenous infusion dose of FCM can reach 1,000 mg, and there are relatively ideal safety and efficacy data (28, 29). FDI can also be administered in high dose, has the advantage of fewer infusions, and is known for its high stability and low risk of infusion reactions (25). Although six intravenous iron formulations are available globally, only five had eligible pharmacoeconomic studies and were included in this analysis. No studies of FMT met the inclusion criteria. The characteristics of the included intravenous iron formulations are presented in Table 1.

**Table 1:** Included intravenous iron formulations and their administration characteristics.

Formulation	Structural characteristics	Dosage and administration	Maximum single dose	Minimum infusion time	Year of approval	Indications
IS	Polynuclear iron (III)-hydroxide core surrounded by sucrose forming a stable complex	Intravenous injection >10 min (200 mg); intravenous infusion 15 min (100 mg); 30 min (200 mg), once weekly	200 mg	30 min	1992	For patients with iron deficiency requiring intravenous iron therapy when oral iron is ineffective or not tolerated, or when oral iron absorption is inadequate
ID	Iron (III)-hydroxide dextran complex	Intravenous infusion 30 min (100–200 mg), 2–3 times/week; total dose infusion 4–6 h (20 mg·kg^−1^)	200 mg/20 mg·kg^−1^	30 min	1991	For patients with iron deficiency who are intolerant to or unresponsive to oral iron therapy
FG	Hydrated ferric oxide complexed with sucrose and chelated with glucose (2:1 ratio) to form a stable macromolecular complex	Recommended dose 62.5 mg; maximum 125 mg; 1 h infusion or 10 min injection	125 mg	1 h	1999	Indicated for treatment of iron deficiency anemia in adults and in children aged ≥6 years with chronic kidney disease receiving hemodialysis who are receiving supplemental epoetin therapy
FCM	Complex of polynuclear ferric hydroxide cores stabilized by carboxymaltose	15 min, with an interval of 1 week between doses	1,000 mg	15 min	2007	For the treatment of iron deficiency in adults and children aged ≥1 year when oral iron is ineffective, not tolerated, or when rapid iron supplementation is clinically required
FDI	Iron (III)-hydroxide complex with isomaltoside (iron isomaltoside complex)	Intravenous injection >2 min (500 mg), up to 3 times/week; intravenous infusion >15 min (<1,000 mg); >30 min (>1,000 mg)	1,500 mg	≤1,000 mg (≥15 min); >1,000 mg (≥30 min)	2009	For patients in whom oral iron is ineffective or not tolerated, or when rapid iron supplementation is clinically indicated
FMT	Superparamagnetic iron oxide colloidal nanoparticles	≥17 s (510 mg: 17 ml); the FDA (2015) recommends an infusion time of ≥15 min	510 mg	≥15 min	2009	Indicated for the treatment of iron deficiency anemia in adults who have intolerance to or an unsatisfactory response to oral iron therapy

IS, iron sucrose; ID, iron dextrose; FG, ferrous gluconate; FCM, ferric carboxymaltose; FDI, ferric derisomaltose; FMT, ferumoxytol.

Although intravenous iron formulations have comparable mechanisms of action, their pharmacological characteristics and dosing regimens differ, which in turn influence clinical outcomes, safety, and overall treatment costs. Through systematic pharmacoeconomic evaluation, the cost-effectiveness of different intravenous iron formulations for treating IDA can be comprehensively assessed. Although several economic studies have been conducted (30–32), most focus on only one or a few formulations and are limited to specific countries or healthcare systems, failing to provide a global perspective. A systematic review can integrate evidence from diverse settings and formulations, offering a more comprehensive understanding of their cost-effectiveness. Therefore, this study aimed to systematically evaluate the economic characteristics of six major intravenous iron formulations for the treatment of IDA worldwide.

## 2 Methods

This systematic review was prospectively registered on PROSPERO (CRD42024544898; https://www.crd.york.ac.uk/prospero/display_record.php?ID=CRD42024544898) and adhered to the Preferred Reporting Items for Systematic Reviews and Meta-Analyses (PRISMA) checklist (33) (Supplementary File 1). Institutional review board approval and participant informed consent were not required for this review because it included only previously published research.

### 2.1 Search strategy

We systematically searched PubMed, Embase, Web of Science, and The Cochrane Library for relevant studies from inception to 30 July 2025. The search strategy included MeSH terms and text words in various combinations to identify IDA patients, six intravenous iron formulations, and economic studies. The search terms included “iron deficiency,” “intravenous iron,” “iron sucrose,” “ferric carboxymaltose,” “ferric derisomaltose,” “iron dextran,” “ferric gluconate,” “ferumoxytol,” “cost effectiveness,” “cost utility,” “cost benefit,” “cost minimization,” “pharmacoeconomics,” and “economic evaluation.” The search strategy was peer-reviewed by a second information specialist. The reference lists of included studies and other published systematic reviews were also manually reviewed for relevant articles. Details of the search strategy are provided in Supplementary File 2.

### 2.2 Study selection

A list of predetermined inclusion and exclusion criteria for study identification is presented in Table 2. Two reviewers (ZX and SM) independently screened titles and abstracts. Similarly, the full text of potentially eligible studies was also retrieved and assessed for eligibility by two independent reviewers (ZX and SM). Any disagreement between the reviewers about the eligibility of a study was resolved through discussion with the third reviewer (QX).

**Table 2:** Inclusion and exclusion criteria for reviewed studies.

Category	Inclusion criteria	Exclusion criteria
Population	Iron deficiency anemia patients, with or without other diseases, with no restrictions on age or gender	None
Intervention	Intravenous iron formulations (iron sucrose, ferric gluconate, iron dextran, ferumoxytol, ferric carboxymaltose, or ferric derisomaltose)	Not applicable
Comparison	Intravenous iron formulations (iron sucrose, ferric gluconate, iron dextran, ferumoxytol, ferric carboxymaltose, or ferric derisomaltose)	Not applicable
Outcomes	Cost, ICER, QALY, ICUR, etc.	None
Study type	Economic evaluation studies: CEA, CUA, CBA, or CMA	BIA
Others	None	Non-English article; letter, review article, conference abstract, protocol, or full text unavailable; irrelevant population, irrelevant comparison, irrelevant intervention, irrelevant types of economic study, or no outcome indicator; duplicate publication

ICER, incremental cost–effect ratio; QALY, quality-adjusted life years; ICUR, incremental cost–utility ratio; CEA, cost-effectiveness analysis; CUA, cost–utility analysis; CBA, cost–benefit analysis; CMA, cost-minimization analysis; BIA, budget impact analysis.

### 2.3 Data extraction

For included studies, data were extracted using a pilot-tested standardized form by one reviewer (ZX) and independently checked for completeness and accuracy by a second reviewer (SM). The extracted data include but are not limited to the following: authors, year of publication, study perspective, country, disease type, intervention drugs, control drugs, cost, outcome indicators, time horizon, economic results, and sensitivity analysis. Reviewers resolved disagreements through discussion, adjudicating with a third reviewer if necessary.

### 2.4 Quality assessment

The quality of all articles included in this study was assessed according to the Consolidated Health Economic Evaluations Reporting Standards (CHEERS) 2022 checklist (34). Two independent reviewers assessed each item in the selected articles and consulted a third reviewer if there was any disagreement. According to the scoring criteria for relevant literature (35), the maximum score for each study was 28 points; each item that fully meets the criteria is scored 1 point, partially meets the criteria is scored 0.5 points, and does not meet the criteria is scored 0 points. Percentage scores were then calculated, and each study was classified into one of four categories: quality scores of ≥85% were classified as excellent quality, 70%–85% as very good quality, 55%–70% as good quality, and <55% as low quality.

### 2.5 Synthesizing data

The review was described according to interventions and the type of economics. Quantitative synthesis of study results was not possible due to the heterogeneity of patients, interventions, cost sources, and study designs.

## 3 Results

### 3.1 Study selection

**Figure 1:**
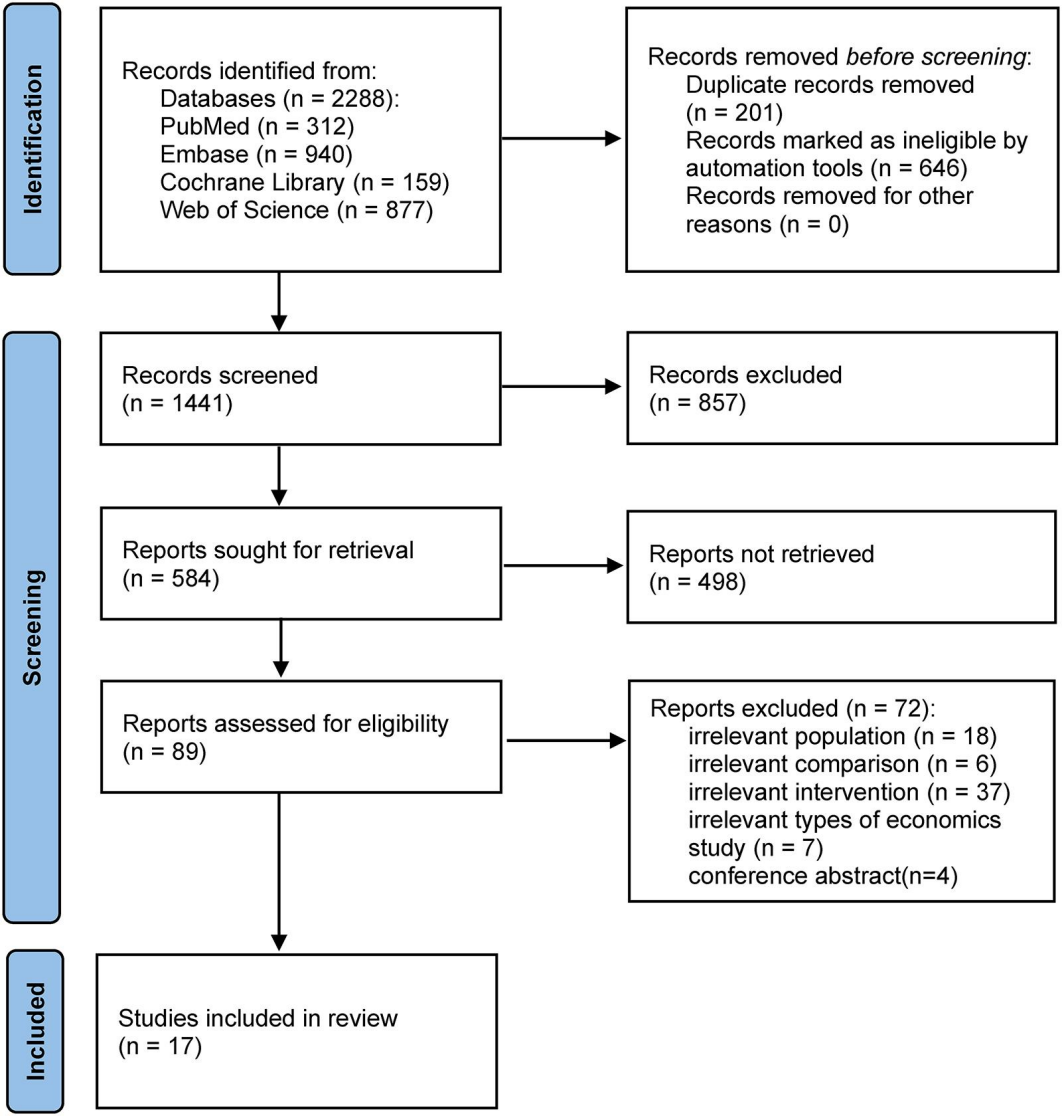
Preferred Reporting Items for Systematic Reviews and Meta-Analyses (PRISMA) flowchart.

Figure 1 shows the flowchart for the selection of the included studies. A total of 2,288 studies were found, and 847 duplicates were excluded from the initial screening. The titles and abstracts of the remaining 1,441 records were screened to exclude 857 articles. The remaining 584 studies were screened, and 498 were excluded because no conference abstracts or reports were found. The full text of the remaining 89 studies was assessed to apply the selection criteria; there were 18 studies with irrelevant populations, 6 with irrelevant comparison, 37 with irrelevant intervention, 7 that did not meet the types of economic study, and 4 conference abstracts. Seventeen studies were finally included in the review.

### 3.2 Main characteristics of economic evaluations

Table 3 summarizes the main characteristics of the economic evaluations. The earliest study was published in 2010. The included studies included five intravenous iron formulations, with the most common being a comparison between FCM and IS.

**Table 3:** Main characteristics of economic evaluations.

Author year	Intervention, comparator	Type of economics	Country	Study perspective	Target population	Indicators of efficacy	Source of efficacy	Costs	Outcome measure	Time horizon and discounting	Sensitivity analysis
Aksan et al., 2021 (36)	FCM vs. FDI, IS	CEA	UK	NHS	IBD with IDA	The percentage of patients achieving a response	NMA	Direct costs (①, ②, ④)	ICER	One cycle of treatment, no discount	DSA
Aksan et al., 2020 (37)			Switzerland	Hospital	IBD with IDA	The percentage of patients achieving a response	NMA	Direct costs (①, ②, ④)	ICER	Not projected beyond a 1 year time horizon, no discount	DSA
Argüelles 2022 (38)	FCM vs. IS		Spain	Healthcare payer	IBD with IDA	The percentage of patients achieving a response	FERGIcor RCT	Direct costs (①, ②, ④)	ICER	One cycle of treatment, no discount	DSA
Basha et al., 2021 (39)			Qatar	Hospital	Adult patients with IDA	clinical test indicators (the change of Hb, TSAT, MCH, and MCV levels)	a cross-sectional study	Direct costs (①, ⑥, ⑨)	ICER	1 year, no discount	—
Pollock et al., 2020 (40)	FCM vs. FDI		UK	Healthcare payer	IDA	The percentage of patients achieving a response	ITC	Direct costs (①, ②)	ICER	One cycle of treatment, no discount	DSA
Szucs et al., 2011 (41)	FCM vs. ID, IS, FG		Switzerland	Healthcare system	Cancer- or chemotherapy-induced anemia	Assuming similar efficacy	—	Direct costs (①, ②)	ICER	Ten weeks, no discount	—
Hu et al., 2022 (42)	FDI vs. IS	CUA	China	Healthcare system and societal	Adult patients with IDA	QALY	PROVIDE RCT	Direct costs (①, ②, ⑩, ⑬)	ICUR	Five years, 5% discounting	DSA, PSA
Iqbal et al., 2024 (43)	FCM vs. FDI		England	Provider, national payer, and societal	IBD with IDA	QALY	PHOSPHARE-IBD RCT	Direct costs (①, ②, ⑧)	ICUR	Five years, 3.5% discounting	DSA, PSA
Zhang et al., 2024 (44)			China	Healthcare system	Adult patients with IDA	QALY	PHOSPHARE-IBD RCT	Direct costs (①, ②, ⑧	ICUR	Five years, 5% discounting	DSA, PSA
Lindgren et al., 2025 (45)			Sweden	National payer	IBD with IDA	QALY	PHOSPHARE-IBD RCT	Direct costs (①, ②, ⑧)	ICUR	Five years, 3% discounting	DSA, PSA
Detlie et al., 2025 (46)			Norway	Payer	IBD with IDA	QALY	PHOSPHARE-IBD RCT	Direct costs (①, ②, ⑧)	ICUR	Five years, 4% discounting	DSA, PSA
Aladham et al., 2023 (47)	FCM vs. IS	CMA	Algeria	Healthcare providers	Pregnant women with IDA	Assuming similar efficacy	—	Direct costs (①, ②, ③, ④) Indirect costs (⑮)	Cost	—	—
Calvet et al., 2016 (48)			Spain	Third-payer	Colon cancer with IDA	Assuming similar efficacy	—	Direct costs (①, ⑤, ⑥) Indirect costs (⑬, ⑭)	Cost	One hospitalization cycle, no discount	DSA, PSA
Calvet et al., 2012 (49)			Spain	Hospital and societal	Digestive conditions with IDA	Assuming similar efficacy	—	Direct costs (①, ②, ⑥, ⑪)	ICER	1 year, no discount	DSA, PSA
Fragoulakis et al., 2012 (50)	FCM vs. IS, ID		Greece	NHS and patients	IDA	Assuming similar efficacy	—	Direct costs (①, ②, ⑦, ⑩, ⑪) Indirect costs (⑯)	Cost	Average length of stay 1 week (inpatients), no discount	DSA
Bhandari 2011 (51)	FCM vs. IS, ID, FDI		UK	Hospital	IDA	Assuming similar efficacy	—	Direct costs (①, ②, ⑨, ⑫)	Cost	—	—
Bager and Dahlerup, 2010 (52)	FCM vs. IS	CBA, CEA	Denmark	Patients and societal	IBD with IDA	Assuming similar efficacy	—	Direct costs (①, ②, ④, ⑫, ⑬) Indirect costs (⑯)	Cost/WTP	—	DSA

① the cost of intravenous iron, ② consumables required to deliver the infusion, ③ iron workup, ④ medical time, ⑤ transfusions, ⑥ hospitalization, ⑦ infusion monitoring, ⑧ phosphate monitoring, phosphate supplementation, ⑨ nursing, ⑩ treatment costs of adverse events, ⑪ travel, ⑫ transport, ⑬ administrative, ⑭ maintenance and cleaning services, ⑮ absenteeism, ⑯ patient income loss.

FCM, ferric carboxymaltose; FDI, ferric derisomaltose; IS, iron sucrose; CEA, cost-effectiveness analysis; NHS, National Health Service; IBD, inflammatory bowel disease; IDA, iron deficiency anemia; NMA, network meta-analysis; ICER, incremental cost–effect ratio; DSA, deterministic sensitivity analysis; Hb, hemoglobin; TSAT, transferrin saturation; MCH, mean corpuscular hemoglobin; MCV, mean corpuscular volume; ITC, indirect treatment comparison; ID: iron dextrose; FG, ferrous gluconate; CUA, cost–utility analysis; QALY, quality-adjusted life years; ICUR, incremental cost–utility ratio; PSA, probabilistic sensitivity analysis; CMA, cost-minimization analysis; CBA, cost–benefit analysis; WTP, willingness to pay.

#### 3.2.1 Country

These studies were conducted in 10 different countries, mostly from European countries, i.e., the UK (*n* = 4), Spain (*n* = 3), Switzerland (*n* = 2), Greece (*n* = 1), Norway (*n* = 1), Sweden (*n* = 1), and Denmark (*n* = 1), and four studies from Asia and Africa, i.e., Algeria (*n* = 1), Qatar (*n* = 1), and China (*n* = 2).

#### 3.2.2 Study perspective

The study perspective plays a crucial role in economic evaluations. Different study perspectives define costs differently, which in turn affects the accuracy and effectiveness of economic evaluations. Determining the study perspective is a prerequisite for evaluating whether an intervention has value and achieves the desired objectives. In the 17 articles, the study perspectives are respectively from healthcare system perspective, healthcare provider’s perspective, payer perspective, patient’s perspective, and societal perspective. Four studies (37, 39, 47, 51) were developed from the healthcare provider’s perspective, the healthcare system perspective was used from three studies (36, 41, 44), the payer perspective was used from six studies (38, 40, 43, 45, 46, 48), and four studies (42, 47, 49, 52) were analyzed from multiple perspectives.

#### 3.2.3 Time horizon and discounting

In pharmacoeconomic evaluations, the time horizon needs to reflect the natural course of the disease reasonably. The onset and progression of IDA vary considerably depending on the underlying cause of iron deficiency. Although hemoglobin levels may improve within months, the rate and extent of recovery largely depend on the underlying cause of iron deficiency and the adequacy of its management. Discount rates enable economic assessments to account for changes in the value of money over time. Most of the studies (36–41, 47–49) included in this review were studied within a 1-year, relatively short time horizon, so most were not discounted. Five studies (42–46) modeled over a 5-year period. Two studies (42, 44) were conducted in China, with benefits and costs discounted at 5% per year, as recommended by the Chinese Pharmaceutical Association (53). In a study (43) conducted in the UK, future costs and impacts were discounted at 3.5% per year, as recommended by the National Institute for Health and Care Excellence (54). The two studies (45, 46) applied annual discount rates of 3% and 4%, respectively.

#### 3.2.4 Indicators of efficacy

Six studies (36–41) conducted cost-effectiveness analyses (CEA). Among them, four defined effectiveness as the proportion of patients achieving a response (Hb normalization or an increase of ≥2 g/dL); one used clinical indices such as Hb, ferritin, transferrin saturation, mean corpuscular Hb, and mean corpuscular volume as efficacy indicators; and one assumed equivalent efficacy. Most studies (36–38, 40, 41) derived efficacy data from RCTs or network meta-analyses (NMA) with high-quality evidence. Two studies (36, 37) used data from the same NMA, which included five RCTs (*n* = 1,143). One study (38) used data from the FERGIcor randomized controlled trial (RCT) involving 485 patients with IBD-associated IDA across 14 countries. Another (40) was based on an indirect treatment comparison, one (41) on a meta-analysis of eight studies comparing intravenous iron with oral or no supplementation, and one (39) on a retrospective cross-sectional study.

Five studies (42–46) performed cost–utility analysis (CUA) using quality-adjusted life years (QALY) as the measure of effectiveness. In one study (42), the model simulated a 5-year time horizon, yielding an undiscounted life expectancy of 4.965 years for both the FDI and IS groups, representing the average life-years over the simulation period rather than actual patient survival. Disease-related health utility values were then applied to estimate QALY. In four studies (43–46), the incidence of hypophosphatemia was derived from the PHOSPHARE-IBD RCT, and patients’ health utility was measured using the Short Form 6-Dimension (SF-6D).

Five studies (47–51) used cost-minimization analysis (CMA), assuming that the interventions were equally effective, and directly compared the cost of intravenous iron formulations.

One study (53) simultaneously compared the healthcare costs of intravenous iron formulations for treating patients with IBD from three economic evaluation perspectives: budget impact analysis (BIA), CEA, and cost–benefit analysis (CBA); only data related to CEA and CBA were included in this study, assuming equal efficacy.

#### 3.2.5 Costs

In cost measurement, it is recommended to prioritize the use of data derived from the national population. If local data are not available, data from other countries should be appropriately adjusted to reflect the characteristics of the national healthcare system. The costs in the studies included in this review were all derived from the home country and therefore did not require data correction. In all the studies included in this review, costs were largely consistent with the research perspective. Costs in the pharmacoeconomic evaluation include direct cost, indirect cost, and intangible cost; the most included direct costs in all the studies were the cost of medication for intravenous iron formulations and the cost of consumables required for infusion, while indirect costs were included for patient absenteeism and loss of productivity due to illness, and no intangible costs were considered in any of the studies.

#### 3.2.6 Target population

A total of seven studies (36–38, 43, 45, 46, 52) were based on patients with IBD with IDA; six studies (39, 40, 42, 44, 50, 51) did not differentiate between disease types; one study (48) was based on patients with colon cancer with IDA; one study (49) was based on patients with digestive conditions with IDA; one study (47) was based on pregnant women with IDA; one study (41) was based on patients with cancer- or chemotherapy-induced anemia.

Although the target populations involved different disease backgrounds, most studies consistently supported the economic advantage of newer intravenous iron formulations. Nevertheless, as more than half of the included studies focused on IBD-related IDA, our findings may be more representative of this subgroup. Formal subgroup analysis was not feasible due to the small number of studies in other disease contexts.

### 3.3 Synthesis of economic results

#### 3.3.1 Results by formulation classification

This review provides an overview of the economic results based on the classification of intravenous iron formulations. The results of the economic evaluation are shown in Table 4.

**Table 4:** The results of the economic evaluations.

Author year	Intervention, comparator	Type of economics	Results	Conclusions	
Aladham et al., 2023 (47)	FCM vs. IS	CMA	The total cost of IS was DZD 69,222 per patient, and DZD 57,452 for FCM, and FCM instead of IS saved DZD 11,769 per patient ($84.74)	FCM is more economical than IS	FCM is more cost-effective than IS
Calvet et al., 2016 (48)			The total cost per patient was €1,827 for FCM and €2,312 for IS. FCM saves €485 per patient compared with IS	Preoperative FCM infusion was less costly than IS infusion and reduced the total cost in patients with IDA with colon cancer.	
Calvet et al., 2012 (49)			In the base analysis, the estimated annual cost of iron infusion was €304 for IS and €274 for FCM. In the societal perspective, non-hospital direct costs were added, amounting to €353.8 for IS and €286.5 for FCM	FCM infusion reduced the costs of iron infusion at a gastrointestinal day-care unit	
Argüelles et al., 2022 (38)		CEA	With a cost of €323 for FCM and €470 for IS, the response rate is 83.8% for FCM and 75.9% for IS, and FCM dominates in comparisons related to increased response rates and cost savings without the need to calculate ICER	FCM is less costly and more effective than IS for the treatment of IDA subsequent to IBD in Spain and therefore was considered dominant	
Bager and Dahlerup, 2010 (52)		CBA, CEA	CEA showed that FCM was more cost-effective than IS, due to fewer outpatient setting visits (range €97–€269, depending on the total dose). CBA showed that the average patient’s “willingness to pay” for a total of iron dose of 1,400 mg was €233 to reduce the number of infusions from 7 to 2 by using FCM rather than IS	Both the CEA and the CBA showed clearly that FCM is a more cost-effective way of giving intravenous iron than IS in IBD patients	
Basha et al., 2021 (39)		CEA	The total cost of FCM treatment was QAR 915, which was significantly lower than that of IS at 1,003 QAR; the changes in clinical indicators were statistically significant only for Hb, RBC, and MCH, with Hb (25.09), RBC (5.64), and MCH (18.71) in the FCM group, and Hb (29.03), RBC (10.92), and MCH in the IS group (23.19), and changes in clinical indicators were higher in the IS group than in the FCM group	ICER indicated that further justifications and decisions need to be made for FCM when using Hb, iron, TSAT, MCH, and MCV levels as surrogate outcomes	IS has higher clinical metric benefit and higher cost than FCM
Aksan et al., 2021 (36)	FCM vs. FDI, IS	CEA	Response rate with FCM, FDI, and IS was 81%, 74%, and 75%, respectively. Total costs with FCM, FDI, and IS were £296, £312, and £503, respectively. FCM was found to be more effective and less costly than both FDI and IS, and there is no need to calculate ICER	FCM is more effective and less costly than FDI and IS	FCM is more effective and less costly than FDI and IS
Aksan et al., 2020 (37)			Response rate with FCM, FDI, and IS was 81%, 74%, and 75%, respectively. Total costs with FCM, FDI, and IS were CHF 461 (€433), CHF 485 (€456), and CHF 608 (€572), respectively. FCM was found to be more effective and less costly than both FDI and IS, and there is no need to calculate ICER	FCM is more effective and less costly than FDI and IS	
Fragoulakis et al., 2012 (50)	FCM vs. IS, ID	CMA	For outpatients, the direct cost of treatment per patient was €198.6 in the FCM group, €627.7 in the IS group, and €510.5 in the ID group. For inpatients, the average cost of treatment was €189.2 in the FCM group, €414.9 in the IS group, and €228.8 in the ID group	FCM is a cost-saving alternative to other intravenous iron formulations, where the cost rank is FCM < ID < IS	
Bhandari 2011 (51)	FCM vs. IS, ID, FDI	CMA	At the 600 mg dose level, IS was £261.61, ID was £221.19, FDI was £170.21, and FCM was £173.23; at the 1,000 mg dose level, IS was £436.02, ID was £253.07, FDI was £231.71, and FCM was £249.63; at the 1,600 mg dose level, £697.63 for IS, £300.89 for ID, £333.41 for FDI and £404.86 for FCM	ID is the least expensive option at the 1,600 mg dose level but has the caveat of a prolonged administration time and requirement for a test dose. At 600 mg and 1,000 mg dose levels, both FDI and FCM are more economical than ID. FDI is less expensive than FCM at all dose levels	
Szucs et al., 2011 (41)	FCM vs. IS, ID, FG	CEA	The 24% increment in Hb response with intravenous iron formulations versus no or oral iron supplementation corresponded to ICER of €1,704, €2,187, €2,455, and €3,686 per additional responder for FCM, ID, IS, and FG, respectively	The economic comparison is FCM, ID, IS, and FG	
Iqbal et al., 2024 (43)	FCM vs. FDI	CUA	The base case analysis from a national payer perspective showed total costs per patient over a 5-year time horizon of £2,414 for FCM and £1,692 for FDI (FCM saved £722 per patient); the micro cost analysis from a provider perspective showed total costs of £1,586 for FCM and £1,310 for FDI (FCM saved 276 per patient); the societal perspective analysis showed that from a national payer perspective, the total cost was £2,692 for FCM and £1,907 for FDI (FCM saved £276 per patient). The QALY for FCM was 2.57, and the QALY for FDI was 2.65 (an increase of 0.075 QALY)	Results showed that FDI improved patient quality of life and reduced direct healthcare expenditure versus FCM in patients with IBD and IDA in England	FDI is more cost-effective than FCM
Pollock et al., 2020 (40)		CEA	The cost of treating patients with FCM and FDI was £637 and £457, respectively, with hematological remission occurring in 79.0% of the FDI group and 70.0% of the FCM	The use of FDI rather than FCM in IDA patients is predominant and will reduce the number of iron infusions, thereby reducing the costs associated with IDA therapy	
Zhang et al., 2024 (44)		CUA	FDI resulted in cost savings of RMB 989 (RMB 4,301 vs. RMB 3,312) and an improvement in quality-adjusted life expectancy of 0.0,705 QALYs (2.50 vs. 2.57) over 5 years	FDI would improve patient quality of life and reduce direct healthcare expenditure versus FCM in patients with IDA in China	
Lindgren et al., 2025 (45)		CUA	Total cost savings with FDI were SEK 14,962 (SEK 68,029 vs. SEK 53,067). FDI also resulted in a 0.076 QALY improvement versus FCM, and FDI was therefore the dominant intervention	Relative to FCM, fewer infusions of FDI were required; there was no need for phosphate monitoring, and disease-related quality of life was improved, while overall costs were reduced	
Detlie et al., 2025 (46)		CUA	Total cost savings with FDI were therefore NOK 9,707 (NOK 35,830 vs. NOK 45,537). FDI also increased quality-adjusted life expectancy by 0.071 QALYs (2.58 vs. 2.65)	FDI resulted in cost savings and improved quality-adjusted life expectancy versus FCM in patients with IDA and IBD in Norway	
Hu et al., 2022 (42)	FDI vs. IS	CUA	From a healthcare system perspective, FDI cost ¥10,380 ($2,480), IS cost ¥8,446 ($2,018); QALY for FDI was 3.814 and IS was 3.736 (a difference of 0.078 QALY), and the ICUR for FDI versus IS was ¥24,901/QALY. From a societal perspective. FDI [¥12,396 ($2,961)] was less costly and more effective compared with IS [¥14,258 ($3,406)]. Using the 2020 Chinese gross domestic product per capita of ¥72,447 ($17,307) as a cost-effectiveness threshold, FDI would be considered cost-effective in China	Both the Chinese healthcare system and society-wide perspectives suggest that FDI is more economically effective than IS	

FCM, ferric carboxymaltose; IS, iron sucrose; Algerian dinar, DZD; CMA, cost-minimization analysis; IDA, iron deficiency anemia; CEA, cost-effectiveness analysis; ICER, incremental cost effect ratio; CBA, cost–benefit analysis; IBD, inflammatory bowel disease; Qatari Rials, QAR; Hb, hemoglobin; RBC, red blood cell; MCH, mean corpuscular hemoglobin; TSAT, transferrin saturation; MCV, mean corpuscular volume; FDI, ferric derisomaltose; ID, iron dextrose; FG, ferrous gluconate; Swiss Franc, CHF; CUA, cost–utility analysis; QALY, quality-adjusted life years; ICUR, incremental cost–utility ratio.

##### 3.3.1.1 FCM vs. IS

Six studies (38, 39, 47–49, 53) compared the economics of FCM and IS. The results of five studies (38, 47–49, 52) indicated an economic advantage of FCM over IS, while one study did not yield a specific conclusion. Three of these studies (47–49) conducted CMA. Aladham et al. (47) compared FCM and IS for treating IDA in pregnant women from a healthcare provider perspective. The study showed that although FCM had higher drug acquisition costs, it required fewer infusions due to its larger single-dose capacity, leading to overall cost savings. Calvet et al. (48) analyzed patients with colon cancer and found that preoperative FCM infusions were associated with lower total costs than IS. Another study (49) involving patients with digestive disorders also demonstrated that higher medication costs for FCM were offset by savings related to fewer infusions and reduced hospital resource utilization. Three studies performed CEA (38, 39, 52). Argüelles et al. (38) analyzed patients with IBD and found that FCM dominated IS, providing higher response rates and overall cost savings without the need to calculate an incremental cost-effectiveness ratio (ICER). The advantage of FCM was mainly due to fewer infusions and reduced use of healthcare resources. Bager and Dahlerup (52) also reported that FCM led to reductions in both direct and indirect costs in CEA, including fewer outpatient visits and lower patient-related costs. In a CBA, patients demonstrated a willingness to pay more for FCM to reduce the number of infusions, confirming its cost-effectiveness. Basha et al. (39) conducted a CEA from the perspective of a public hospital in Qatar. The study found that while IS achieved slightly greater hematologic improvement, its total treatment cost was significantly higher, suggesting that FCM may still be the more cost-effective option overall.

##### 3.3.1.2 FCM vs. IS, ID

Fragoulakis et al. (50) compared FCM, IS, and ID in both inpatients and outpatients using CMA. The results suggested that FCM was the most cost-saving option, followed by ID and IS.

##### 3.3.1.3 FCM vs. ID, IS, FG

Szucs et al. (41) examined the potential health economic impact of FCM, ID, IS, and FG in patients with cancer- or chemotherapy-induced anemia. From a healthcare system perspective, FCM showed the most favorable cost-effectiveness profile, followed by ID, IS, and FG.

##### 3.3.1.4 FCM vs. IS, ID, FDI

Bhandari (51) conducted a CMA comparing FCM, IS, ID, and FDI at different dose levels from a hospital perspective. Both FDI and FCM were more economical than ID at moderate doses. Although ID became cheaper at the highest dose, it required longer administration time and a test dose. FDI remained the least costly option across all dose levels.

##### 3.3.1.5 FCM vs. IS, FDI

Aksan et al. (36, 37) compared FCM, IS, and FDI in two studies based in Switzerland and the UK. Both found that FCM was more effective and less costly than FDI and IS. The dominance of FCM was attributed to higher response rates and reduced treatment costs without requiring ICER calculation.

##### 3.3.1.6 FCM vs. FDI

Five studies (40, 43–46) compared FCM and FDI, showing that FDI was more effective and less costly than FCM. Four studies (43–46) from different countries conducted CUA using efficacy data from the PHOSPHARE-IBD RCT. Results showed that FCM was associated with a higher incidence of hypophosphatemia, which negatively affected quality of life and added management costs. FDI offered overall cost savings primarily due to fewer infusions. Pollock et al. (40) also demonstrated that FDI achieved higher remission rates and was more cost-effective than FCM.

##### 3.3.1.7 IS vs. FDI

Hu et al. (42) compared IS and FDI using patient-level modeling from healthcare system and societal perspectives. The analysis showed that FDI was more cost-effective than IS, providing higher QALY at lower or comparable total costs.

#### 3.3.2 Results by economic model type

The economic results were summarized based on the types of economic models employed in the included studies.

In CUA, FDI demonstrated more favorable cost-effectiveness profiles compared with FCM and IS. In CMA, FCM was consistently identified as the most economical option relative to IS and ID. In CBA, FCM also showed superior economic performance compared with IS. In CEA, FCM was generally more cost-effective than ID, IS, and FG, while FDI demonstrated economic advantages over IS. No direct or consistent comparative evidence was available between FCM and FDI.

### 3.4 Quality assessment

Economic assessment integrates multiple factors of disease intervention, including effectiveness, safety, and cost, and involves many methodological steps. Errors or inconsistencies at any stage may affect the final outcomes. Therefore, assessing the methodological quality of included studies is essential in pharmacoeconomic systematic reviews to ensure reliability, minimize bias, and enhance scientific rigor. Quality assessment also helps identify high-quality evidence, improving the validity, applicability, and policy relevance of the conclusions.

**Figure 2:**
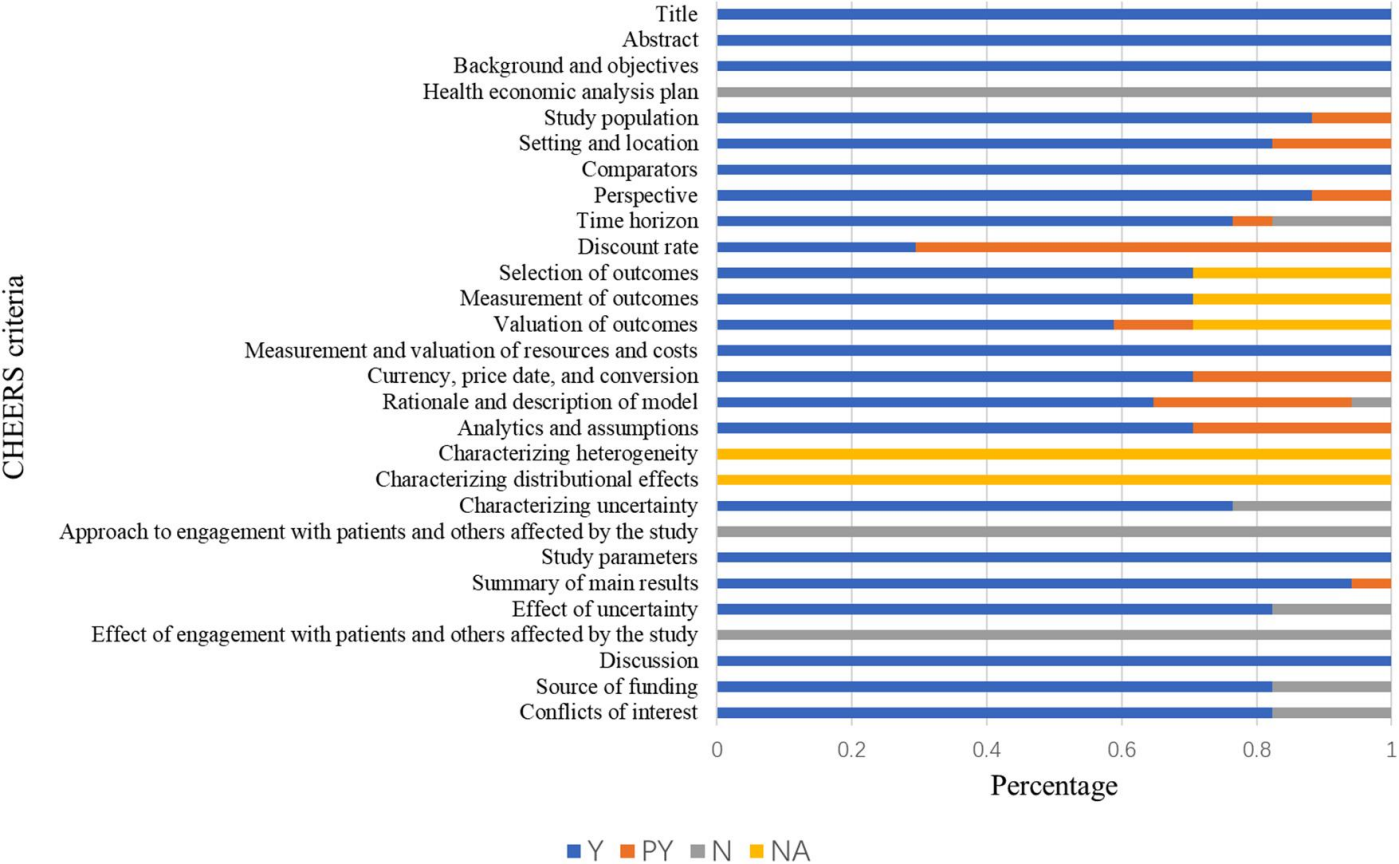
The results of the quality assessment of the studies using the CHEERS 2022 checklist.

Figure 2 shows the quality assessment results based on the CHEERS 2022 checklist. The mean study quality score was 0.82 ± 0.06 (Supplementary File 3), indicating overall high quality. Terms 11–13 were not applicable to some studies due to irrelevance to cost analysis, and Terms 18 and 19 were not applicable to all studies. None of the studies reported a health economic analysis plan (Term 4); although still in its early stage, authors should indicate whether such a plan was developed and how it can be accessed. Similarly, none of the studies reported patient or stakeholder involvement (Terms 21 and 25). Six studies were rated as excellent (36, 42, 44–46, 49), ten as very good (37–40, 43, 47, 48, 50–52), and one as good (52). Overall, most studies met the CHEERS 2022 criteria, indicating generally good methodological quality.

### 3.5 Sensitivity analyses

In pharmacoeconomic evaluations, variability may arise due to variations in regions and contexts (such as differences in treatment regimens, healthcare access, payment methods, etc.) or patient heterogeneity, and these variabilities cannot be eliminated (47). Researchers should conduct a comprehensive analysis of the various sources of uncertainty in the process of economic evaluation, and both deterministic and probabilistic sensitivity analysis results should be reported in the economic evaluation. Therefore, this study provides an overview of sensitivity analyses of the included studies.

Sensitivity analyses were performed in all but four studies (39, 41, 47, 51). The studies included in this review used deterministic sensitivity analysis and probabilistic sensitivity analysis. Six studies (36–38, 40, 50, 52) used only deterministic sensitivity analysis, and seven studies (42–46, 48, 49) used both deterministic sensitivity analysis and probabilistic sensitivity analysis. Most studies examined the effect on the economic outcome in sensitivity analysis by varying the following parameters: the 95% CI of the ratio, baseline body weight, baseline Hb level, cost of the drugs, or using the Ganzoni formula.

## 4 Discussion

This systematic review synthesized evidence from 17 economic evaluations comparing intravenous iron formulations for the treatment of IDA. Overall, FCM and FDI were more likely to be cost-effective than other formulations. The economic benefits of intravenous formulations mainly arise from fewer required infusions, reduced hospitalization costs, and improved quality of life. Quantitative synthesis was not feasible due to heterogeneity in study design and cost structures.

This review has several notable strengths. To our knowledge, it is the first systematic review evaluating the economic evidence of six intravenous iron formulations for IDA. It applied clear inclusion and exclusion criteria and a comprehensive search strategy across major databases, minimizing the risk of missing relevant studies. The relevance, reliability, and methodological quality of included studies were assessed using a validated appraisal tool by two independent reviewers. The protocol was prospectively registered in PROSPERO to reduce duplication and reporting bias, and the review was conducted in accordance with PRISMA 2020 to ensure transparency and rigor.

FCM and FDI are both high-dose intravenous iron formulations that enable rapid iron supplementation, improve patient compliance, and are more likely to be cost-effective than other formulations by reducing the number of infusions, visits, and associated costs, as well as lowering the need for transfusions and erythropoietin use. Because of their similar pharmacological profiles, comparisons between FCM and FDI have attracted considerable attention; however, existing studies report inconsistent findings, some favoring FCM and others FDI, suggesting that their overall economic difference may be minimal. These discrepancies likely arise from variations in study design, efficacy endpoints, cost components, and patient characteristics. Further large-scale comparative studies are warranted to establish their relative cost-effectiveness.

Safety-related costs should be adequately considered in economic evaluations. Adverse reactions associated with intravenous iron formulations can influence overall treatment costs through additional monitoring, management interventions, or treatment discontinuation. Most included studies did not fully account for the impact of safety on economic results. Only five studies included the costs of treating adverse events, and several reported higher expenditures in the FCM group due to hypophosphatemia. Evidence from RCTs (55) indicates that hypophosphatemia occurs more frequently with FCM than with FDI, leading to additional costs for serum phosphate monitoring and supplementation. Therefore, future evaluations involving FCM should incorporate these costs to improve accuracy.

Heterogeneity among the included studies represents an important limitation of this review. Variations in data sources, clinical efficacy inputs, underlying disease models, patient characteristics, and modeling approaches can all introduce bias and affect the comparability of economic outcomes. For instance, some studies assumed equivalent efficacy between formulations, while others focused primarily on inflammatory bowel disease-associated anemia, in which chronic inflammation and impaired iron absorption may increase treatment frequency and costs compared with anemia in pregnancy or oncology. Differences in the methods used to estimate iron requirements, including the application of the Ganzoni formula, further contribute to variability. In addition, infusion-related hypersensitivity reactions, although generally comparable across formulations, may be influenced by the total number of infusions required to achieve the target iron dose. Furthermore, differences in study perspectives and regional healthcare contexts restrict the transferability of results. The included evaluations were conducted in countries with diverse healthcare systems and cost structures, meaning that findings from one setting may not directly apply to another. Hence, conclusions from this review should be interpreted with caution and within the context of specific health system perspectives.

This review included pharmacoeconomic evaluations of five intravenous iron formulations. Future pharmacoeconomic research should prioritize real-world cost analyses using country-specific data, incorporate patient-reported outcomes and safety-related expenditures, and explore head-to-head comparisons through unified modeling frameworks. Such evidence will provide a more robust foundation for optimizing resource allocation and guiding clinical and policy decisions regarding intravenous iron therapy worldwide.

## 5 Conclusions

In summary, this review systematically evaluates the economic characteristics of the six main intravenous iron formulations used to treat IDA. According to this systematic review, FCM and FDI may be more cost-effective therapeutic options than other intravenous iron formulations. Current evidence suggests that the efficacy of FDI is better than IS, and the economic ranking of the four intravenous iron formulations can be summarized as FCM, ID, IS, and FG. Further studies are needed to justify the economic comparison between FCM and FDI.

## Data Availability

The original contributions presented in the study are included in the article/Supplementary Material, further inquiries can be directed to the corresponding authors.
