# Supplementary material for: Economic evaluation of intravenous iron formulations for patients with iron deficiency anemia: a systematic review
Source: Front Health Serv. 2025 Nov 19;5:1690519. doi: 10.3389/frhs.2025.1690519 (PMC12672858; doi:10.3389/frhs.2025.1690519)
Supplement: Supplementary file 2 [file Datasheet2.docx]

**Supplementary files 2. Search strategy.**

PubMed:

Embase:

#16. #3 AND #6 AND #15

#15. #7 OR #8 OR #9 OR #10 OR #11 OR #12 OR #13 OR #14

#14. 'ferum oxytol':ti,ab,kw OR 'oxide, ferrosoferric':ti,ab,kw OR 'ferriferrous oxide':ti,ab,kw OR 'oxide, ferriferrous':ti,ab,kw OR 'feraheme':ti,ab,kw OR 'ferumoxytol':ti,ab,kw

#13. 'ferric gluconate':ab,kw,ti OR 'sodium ferrigluconate':ab,kw,ti OR 'ferrlecit 100':ab,kw,ti OR 'ferlecit':ab,kw,ti OR 'ferlixit':ab,kw,ti OR 'ferrigluconate sodium':ab,kw,ti OR 'ferrlecit':ab,kw,ti OR 'nulecit':ab,kw,ti OR 'gluconate ferric sodium':ab,kw,ti OR 'sodium ironiiigluconate':ab,kw,ti OR 'iron iii gluconate':ab,kw,ti

#12. 'dextran-iron':ti,ab,kw OR 'dextran iron':ti,ab,kw OR 'iron dextran':ti,ab,kw OR 'iron-dextran':ti,ab,kw OR 'ferridextran':ti,ab,kw OR 'infed':ti,ab,kw OR 'norferan':ti,ab,kw OR 'feosol':ti,ab,kw OR 'hematran':ti,ab,kw OR 'icar':ti,ab,kw OR 'imfergen':ti,ab,kw OR 'imferon':ti,ab,kw OR 'imposil':ti,ab,kw OR 'dextrofer':ti,ab,kw OR 'dexferrum':ti,ab,kw OR 'imperon':ti,ab,kw OR 'cosmofer':ti,ab,kw

#11. 'iron isomaltoside':ti,ab,kw OR 'iron isomaltoside 1000':ti,ab,kw OR 'ferric derisomaltose':ti,ab,kw OR 'isomaltose iron':ti,ab,kw OR 'isomaltose, ferric complex':ti,ab,kw OR 'iron isomaltose':ti,ab,kw OR 'monofer':ti,ab,kw OR 'monoferric':ti,ab,kw

#10. 'ferric carboxymaltose':ti,ab,kw OR fcm:ti,ab,kw OR 'iron carboxymaltose':ti,ab,kw OR 'iron dextri-maltose':ti,ab,kw OR ferinject:ti,ab,kw OR 'vit-45':ab,kw,ti OR 'vit 45':ti,ab,kw OR injectafer:ti,ab,kw

#9. 'iron sucrose':ab,kw,ti OR 'saccharated ferric oxide':ab,kw,ti OR 'ferric saccharate':ab,kw,ti OR 'iron-saccharate':ab,kw,ti OR 'ferri-saccharate':ab,kw,ti OR 'iron saccharate':ab,kw,ti OR 'ferri saccharate':ab,kw,ti OR 'iron oxide saccharated':ab,kw,ti OR 'iron(iii)-hydroxide sucrose complex':ab,kw,ti OR 'venofer':ab,kw,ti OR 'hippiron':ab,kw,ti

#8. 'intravenous iron':ab,kw,ti OR 'iv iron':ab,kw,ti OR 'iron infusions':ab,kw,ti OR 'parenteral iron':ab,kw,ti

#7. 'iron saccharate'/exp OR 'ferric carboxymaltose'/exp OR 'iron isomaltose'/exp OR 'iron dextran'/exp OR 'ferric gluconate'/exp OR 'ferumoxytol'/exp OR 'iron isomaltoside'/exp

#6. #4 OR #5

#5. 'iron-deficiency':ti,ab,kw OR 'iron deficiency':ti,ab,kw OR 'iron deficiencies':ti,ab,kw OR 'sideropenia':ti,ab,kw OR 'sideropenias':ti,ab,kw OR 'hypoferritinemia':ti,ab,kw OR 'hypoferritinemias':ti,ab,kw OR 'asiderosis':ti,ab,kw OR 'fe deficiency':ti,ab,kw OR 'fe ion deficiency':ti,ab,kw OR 'fe (iii) deficiency':ti,ab,kw OR 'sideropaenia':ti,ab,kw

#4. 'iron deficiency'/exp

#3. #1 OR #2

#2. 'cost-effectiveness':ab,kw,ti OR 'cost effectiveness':ab,kw,ti OR cost*:ab,kw,ti OR 'cost efficiency':ab,kw,ti OR 'cea':ab,kw,ti OR 'cost-benefit':ab,kw,ti OR 'cost benefit':ab,kw,ti OR 'cost-utility':ab,kw,ti OR 'cost utility':ab,kw,ti OR economic*:ab,kw,ti OR 'cua':ab,kw,ti OR 'cost minimization':ab,kw,ti OR 'cost-minimization':ab,kw,ti OR affordabilit*:ab,kw,ti OR 'pricing':ab,kw,ti OR pharmacoeconomic*:ab,kw,ti OR 'cma':ab,kw,ti OR 'cba':ab,kw,ti OR 'pharmaco-economic analysis':ab,kw,ti OR 'pharmaco-economic evaluation':ab,kw,ti OR 'pharmaco-economics':ab,kw,ti

#1. 'cost effectiveness analysis'/exp OR 'cost utility analysis'/exp OR 'cost benefit analysis'/exp OR 'cost minimization analysis'/exp OR 'pharmacoeconomics'/exp OR 'economic evaluation'/exp

The Cochrane Library

#1 MeSH descriptor: [Cost-Effectiveness Analysis] explode all trees

#2 MeSH descriptor: [Cost-Benefit Analysis] explode all trees

#3 MeSH descriptor: [Costs and Cost Analysis] explode all trees

#4 MeSH descriptor: [Economics, Pharmaceutical] explode all trees

#5 (Cost-Effectiveness):ti,ab,kw OR (Cost Effectiveness):ti,ab,kw OR (cost*):ti,ab,kw OR (cost efficiency analysis):ti,ab,kw OR (Cost-Benefit):ti,ab,kw

#6 (Cost Benefit):ti,ab,kw OR (Cost-Utility):ti,ab,kw OR (Cost Utility):ti,ab,kw OR (economic*):ti,ab,kw OR (Affordabilit*):ti,ab,kw

#7 (Pricing):ti,ab,kw OR (Cost Minimization):ti,ab,kw OR (Cost-Minimization):ti,ab,kw OR (pharmacoeconomic*):ti,ab,kw OR (CEA):ti,ab,kw

#8 (CUA):ti,ab,kw OR (CBA):ti,ab,kw OR (CMA):ti,ab,kw

#9 #1 OR #2 OR #3 OR #4 OR #5 OR #6 OR #7 OR #8

#10 MeSH descriptor: [Iron Deficiencies] explode all trees

#11 (Iron-Deficiency):ti,ab,kw OR (Iron Deficiency):ti,ab,kw OR (Iron Deficiencies):ti,ab,kw OR (Sideropenia):ti,ab,kw OR (Sideropenias):ti,ab,kw

#12 (Hypoferritinemia):ti,ab,kw OR (Hypoferritinemias):ti,ab,kw

#13 #10 OR #11 OR #12

#14 MeSH descriptor: [Ferric Oxide, Saccharated] explode all trees

#15 MeSH descriptor: [Iron-Dextran Complex] explode all trees

#16 MeSH descriptor: [Ferrosoferric Oxide] explode all trees

#17 (iron sucrose):ti,ab,kw OR (Saccharated Ferric Oxide):ti,ab,kw OR (Ferric Saccharate):ti,ab,kw OR (Iron-Saccharate):ti,ab,kw OR (Ferri-Saccharate):ti,ab,kw

#18 (Iron Saccharate):ti,ab,kw OR (Ferri Saccharate):ti,ab,kw OR (Iron Oxide (Saccharated)):ti,ab,kw OR (Venofer):ti,ab,kw OR (Hippiron):ti,ab,kw

#19 (Ferric Carboxymaltose):ti,ab,kw OR (FCM):ti,ab,kw OR (iron carboxymaltose):ti,ab,kw OR (iron dextri-maltose):ti,ab,kw OR (Ferinject):ti,ab,kw

#20 (VIT-45):ti,ab,kw OR (VIT 45):ti,ab,kw OR (injectafer):ti,ab,kw OR (iron isomaltoside):ti,ab,kw OR (ferric derisomaltose):ti,ab,kw

#21 (isomaltose iron):ti,ab,kw OR (isomaltose, ferric complex):ti,ab,kw OR (iron isomaltose):ti,ab,kw OR (Monofer):ti,ab,kw OR (Monoferric):ti,ab,kw

#22 (Dextran-Iron):ti,ab,kw OR (Dextran Iron):ti,ab,kw OR (Iron Dextran):ti,ab,kw OR (Iron-Dextran):ti,ab,kw OR (Ferridextran):ti,ab,kw

#23 (InFed):ti,ab,kw OR (Norferan):ti,ab,kw OR (Feosol):ti,ab,kw OR (Hematran):ti,ab,kw OR (Icar):ti,ab,kw

#24 (Imfergen):ti,ab,kw OR (Imferon):ti,ab,kw OR (Imposil):ti,ab,kw OR (Dextrofer):ti,ab,kw OR (Dexferrum):ti,ab,kw

#25 (Imperon):ti,ab,kw OR (CosmoFer):ti,ab,kw OR (ferric gluconate):ti,ab,kw OR (sodium iron(III)gluconate):ti,ab,kw OR (sodium ferrigluconate):ti,ab,k

#26 (Ferrlecit 100):ti,ab,kw OR (ferlecit):ti,ab,kw OR (ferlixit):ti,ab,kw OR (ferrigluconate sodium):ti,ab,kw OR (ferrlecit):ti,ab,kw

#27 (nulecit):ti,ab,kw OR (gluconate ferric sodium):ti,ab,kw OR (iron (iii) gluconate):ti,ab,kw OR (Ferum-Oxytol):ti,ab,kw OR (ferumoxytol):ti,ab,kw

#28 (Oxide, Ferrosoferric):ti,ab,kw OR (Ferriferrous Oxide):ti,ab,kw OR (Oxide, Ferriferrous):ti,ab,kw OR (Feraheme):ti,ab,kw OR (Feru-moxytol):ti,ab,kw

#29 (Intravenous iron):ti,ab,kw OR (iv iron):ti,ab,kw OR (iron infusions):ti,ab,kw OR (parenteral iron):ti,ab,kw

#30 #14 OR #15 OR #16 OR #17 OR #18 OR #19 OR #20 OR #21 OR #22 OR #23 OR #24 OR #25 OR #26 OR #27 OR #28 OR #29

#31 #9 AND #13 AND #30

Web of science
