# Supplementary material for: Economic evaluation of intravenous iron formulations for patients with iron deficiency anemia: a systematic review
Source: Front Health Serv. 2025 Nov 19;5:1690519. doi: 10.3389/frhs.2025.1690519 (PMC12672858; doi:10.3389/frhs.2025.1690519)
Supplement: Supplementary file 3 [file Datasheet3.pdf]

Supplementary files 3. CHEERS 2022 checklist.

**Author Year**	**1**	**2**	**3**	**4**	**5**	**6**	**7**	**8**	**9**	**10**	**11**	**12**	**13**	**14**	**15**	**16**	**17**	**18**	**19**	**20**	**21**	**22**	**23**	**24**	**25**	**26**	**27**	**28**	**Scores**	**Quality categorie**
**Aksan 2021(36)**	Y	Y	Y	N	Y	Y	Y	Y	Y	PY	Y	Y	Y	Y	Y	Y	Y	NA	NA	Y	N	Y	Y	Y	N	Y	Y	Y	87.50%	excellent quality
**Aksan 2020(37)**	Y	Y	Y	N	Y	Y	Y	PY	Y	PY	Y	Y	Y	Y	Y	PY	PY	NA	NA	Y	N	Y	PY	Y	N	Y	Y	Y	80.36%	very good quality
**Arias 2022(38)**	Y	Y	Y	N	Y	Y	Y	PY	Y	PY	Y	Y	Y	Y	Y	PY	Y	NA	NA	Y	N	Y	Y	Y	N	Y	Y	Y	83.93%	very good quality
**Basha 2021(39)**	Y	Y	Y	N	Y	PY	Y	Y	Y	PY	Y	Y	Y	Y	PY	Y	Y	NA	NA	N	N	Y	Y	N	N	Y	Y	Y	76.79%	very good quality
**Pollock 2020(40)**	Y	Y	Y	N	PY	Y	Y	Y	PY	PY	Y	Y	PY	Y	Y	Y	Y	NA	NA	Y	N	Y	Y	Y	N	Y	Y	Y	82.14%	very good quality
**Szucs 2011(41)**	Y	Y	Y	N	Y	Y	Y	Y	Y	PY	Y	Y	Y	Y	PY	N	PY	NA	NA	N	N	Y	Y	N	N	Y	Y	N	69.64%	good quality
**Hu 2022(42)**	Y	Y	Y	N	Y	Y	Y	Y	Y	Y	Y	Y	Y	Y	Y	Y	PY	NA	NA	Y	N	Y	Y	Y	N	Y	Y	Y	87.50%	excellent quality
**Iqbal 2024(43)**	Y	Y	Y	N	Y	Y	Y	Y	Y	Y	Y	Y	PY	Y	Y	Y	Y	NA	NA	Y	N	Y	Y	N	N	Y	Y	N	80.36%	very good quality
**Zhang 2024(44)**	Y	Y	Y	N	Y	Y	Y	Y	Y	Y	Y	Y	Y	Y	Y	Y	Y	NA	NA	Y	N	Y	Y	Y	N	Y	Y	Y	89.29%	excellent quality
**Lindgren 2025(45)**	Y	Y	Y	N	Y	Y	Y	Y	Y	Y	Y	Y	Y	Y	Y	Y	Y	NA	NA	Y	N	Y	Y	Y	N	Y	Y	Y	89.29%	excellent quality
**Detlie 2025(46)**	Y	Y	Y	N	Y	Y	Y	Y	Y	Y	Y	Y	Y	Y	Y	Y	Y	NA	NA	Y	N	Y	Y	Y	N	Y	Y	Y	89.29%	excellent quality
**Aladham 2023(47)**	Y	Y	Y	N	Y	PY	Y	Y	N	PY	NA	NA	NA	Y	PY	PY	Y	NA	NA	N	N	Y	Y	Y	N	Y	Y	Y	75.00%	very good quality
**Calvet 2016(48)**	Y	Y	Y	N	Y	Y	Y	Y	Y	PY	NA	NA	NA	Y	PY	PY	Y	NA	NA	Y	N	Y	Y	Y	N	Y	N	Y	80.36%	very good quality
**Calvet 2012(49)**	Y	Y	Y	N	Y	Y	Y	Y	Y	PY	NA	NA	NA	Y	Y	Y	Y	NA	NA	Y	N	Y	Y	Y	N	Y	Y	Y	87.50%	excellent quality
**Fragoulakis 2012(50)**	Y	Y	Y	N	Y	Y	Y	Y	Y	PY	NA	NA	NA	Y	PY	Y	PY	NA	NA	Y	N	Y	Y	Y	N	Y	Y	Y	83.93%	very good quality
**Bhandari 2011(51)**	Y	Y	Y	N	PY	PY	Y	Y	N	PY	NA	NA	NA	Y	Y	Y	Y	NA	NA	N	N	Y	Y	Y	N	Y	N	Y	73.21%	very good quality
**Bager 2010(52)**	Y	Y	Y	N	Y	Y	Y	Y	N	PY	Y	Y	Y	Y	Y	PY	PY	NA	NA	Y	N	Y	Y	Y	N	Y	N	N	73.21%	very good quality
